# Antibiotic Resistance in Animal and Environmental Samples Associated with Small-Scale Poultry Farming in Northwestern Ecuador

**DOI:** 10.1128/mSphere.00021-15

**Published:** 2016-02-10

**Authors:** Nikolay P. Braykov, Joseph N. S. Eisenberg, Marissa Grossman, Lixin Zhang, Karla Vasco, William Cevallos, Diana Muñoz, Andrés Acevedo, Kara A. Moser, Carl F. Marrs, Betsy Foxman, James Trostle, Gabriel Trueba, Karen Levy

**Affiliations:** a Program in Population Biology, Ecology and Evolution, Emory University, Atlanta, Georgia, USA; b Department of Epidemiology, University of Michigan, Ann Arbor, Michigan, USA; c Department of Epidemiology & Biostatistics, Michigan State University, East Lansing, Michigan, USA; d Department of Microbiology and Molecular Genetics, Michigan State University, East Lansing, Michigan, USA; e Instituto de Microbiología, Universidad San Francisco de Quito, Quito, Ecuador; f Universidad Central del Ecuador, Quito, Ecuador; g Department of Anthropology, Trinity College, Hartford, Connecticut, USA; h Department of Environmental Health, Emory University, Atlanta, Georgia, USA; University of Minnesota

**Keywords:** antibiotic resistance, epidemiology, microbial ecology

## Abstract

The effects of animal agriculture on the spread of antibiotic resistance (AR) are cross-cutting and thus require a multidisciplinary perspective. Here we use ecological, epidemiological, and ethnographic methods to examine populations of *Escherichia coli* circulating in the production poultry farming environment versus the domestic environment in rural Ecuador, where small-scale poultry production employing nontherapeutic antibiotics is increasingly common. We sampled 262 “production birds” (commercially raised broiler chickens and laying hens) and 455 “household birds” (raised for domestic use) and household and coop environmental samples from 17 villages between 2010 and 2013. We analyzed data on zones of inhibition from Kirby-Bauer tests, rather than established clinical breakpoints for AR, to distinguish between populations of organisms. We saw significantly higher levels of AR in bacteria from production versus household birds; resistance to either amoxicillin-clavulanate, cephalothin, cefotaxime, and gentamicin was found in 52.8% of production bird isolates and 16% of household ones. A strain jointly resistant to the 4 drugs was exclusive to a subset of isolates from production birds (7.6%) and coop surfaces (6.5%) and was associated with a particular purchase site. The prevalence of AR in production birds declined with bird age (*P* < 0.01 for all antibiotics tested except tetracycline, sulfisoxazole, and trimethoprim-sulfamethoxazole). Farming status did not impact AR in domestic environments at the household or village level. Our results suggest that AR associated with small-scale poultry farming is present in the immediate production environment and likely originates from sources outside the study area. These outside sources might be a better place to target control efforts than local management practices.

**IMPORTANCE** In developing countries, small-scale poultry farming employing antibiotics as growth promoters is being advanced as an inexpensive source of protein and income. Here, we present the results of a large ecoepidemiological study examining patterns of antibiotic resistance (AR) in *E. coli* isolates from small-scale poultry production environments versus domestic environments in rural Ecuador, where such backyard poultry operations have become established over the past decade. Our previous research in the region suggests that introduction of AR bacteria through travel and commerce may be an important source of AR in villages of this region. This report extends the prior analysis by examining small-scale production chicken farming as a potential source of resistant strains. Our results suggest that AR strains associated with poultry production likely originate from sources outside the study area and that these outside sources might be a better place to target control efforts than local management practices.

## Abstract (precis)

In developing countries, small-scale poultry farming employing antibiotics as growth promoters is being advanced as an inexpensive source of protein and income. Here, we present the results of a large ecoepidemiological study examining patterns of antibiotic resistance (AR) in *E. coli* isolates from small-scale poultry production environments versus domestic environments in rural Ecuador, where such backyard poultry operations have become established over the past decade. Our previous research in the region suggests that introduction of AR bacteria through travel and commerce may be an important source of AR in villages of this region. This report extends the prior analysis by examining small-scale production chicken farming as a potential source of resistant strains. Our results suggest that AR strains associated with poultry production likely originate from sources outside the study area and that these outside sources might be a better place to target control efforts than local management practices.

## INTRODUCTION

Antibiotic resistance (AR) is a growing public health concern in the United States (1) and globally (2). The overuse and misuse of antibiotics in human medicine (3, 4) and in animal agriculture, where the vast majority of antimicrobials are used (5), contribute to the evolution and spread of antibiotic-resistant pathogens (6). Farmed animals and the broader environment can serve as reservoirs of AR genes that can be exchanged across species (7–10). Numerous resistance genes in human pathogens have environmental origins (11), and the environmental resistome is enriched and mobilized when soil and water are contaminated with runoff from farms (12) or with antibiotic residues (5, 13). Multiple pathways link AR in these reservoirs to human health; epidemiological studies going back to the 1970s show an association between antibiotic use on farms and colonization with livestock-associated strains in workers (14) and surrounding communities (15).

Although restricted in developed countries (16), the nontherapeutic use of antibiotics in animal husbandry is increasing in many developing countries, fueled by the rapid growth of poultry production that relies heavily on antibiotics for growth promotion (17, 18). Backyard poultry farming is promoted as an economic development and nutrition supplementation strategy in Africa, Asia, and Latin America (19, 20). Given the sustained global increase in poultry and concurrent use of agricultural antibiotics, there is a need for studies examining connections between animals in intensive production and the surrounding environment and potential human health impacts, especially where animals are raised in close proximity to human communities.

In this report, we compare and examine AR data from *Escherichia coli* populations circulating in poultry production versus domestic environments in a field site in rural Northwest Ecuador. Our previous research suggests that introduction of AR bacteria from outside sources may be an important source of AR in villages of this region (21). This report extends the prior analysis by more closely analyzing one potential source of AR, small-scale production chicken farming.

## RESULTS

### Quantitative characterization of poultry production.

Monthly census surveys showed wide variability in the number of production birds raised over time. Of the 17 villages for which monthly data were available, 15 were categorized based on the maximum number of birds recorded by our surveillance surveys (i.e., the intensity of production in the villages) during the study period (high intensity, >500 birds, *n* = 4; medium intensity, 150 to 500 birds, *n* = 7; low intensity, <150 birds, *n* = 4; no data available, *n* = 2; see Fig. S1 in the supplemental material). Poultry production was intermittent in all of the villages, with periods when no broilers were raised. In high-intensity villages, the size of flocks rarely exceeded 500 during the surveys. At the time of sampling visits, birds were actively farmed in 10 villages.

10.1128/mSphere.00021-15.2 Figure S1  Monthly census of production birds (broilers and laying hens) in 15 study sites. Data for the sites have been split into groups based on the maximum number of birds recorded at any sampling visit. Discontinuities in the lines represent missing data not collected due to logistical limitations. Download Figure S1, DOCX file, 0.01 MB. Copyright © 2016 Braykov et al. 2016 Braykov et al. This content is distributed under the terms of the Creative Commons Attribution 4.0 International license.

### Qualitative characterization of poultry production.

Ethnographic interviews confirmed that poultry production varied widely over time and in scale: while some households periodically maintained flocks of ~10 birds, others housed up to several hundred. Most backyard coops were located within 50 m of houses in raised open structures or directly below the family home. Two villages in the region had large facilities (for housing up to 1,500 birds of multiple ages) built with foreign and local government aid, located away from other houses, and run cooperatively by a group of community members.

Ethnography also confirmed that poultry production in this region is highly intermittent and characterized by “boom and bust” cycles. Development projects commonly provide training, birds, and initial supplies. However, once outside support is withdrawn, production may become unsustainable due to lack of reinvestment in the flock, outbreaks of diseases, and other factors negatively impacting yield. Another major driver of variability in poultry farming is the fluctuating demand inherent in the market. This is particularly true in remote communities where markets are seasonal or episodic and driven by demand during holidays.

Additional ethnographic details about poultry production in the region, as well as results of the chemical analysis of poultry feed, can be found in the supplemental material.

### Sample characterization and susceptibility breakpoints.

Counts of isolates and samples positive for *E. coli* included in the analysis are shown in Table 1. Household birds (*n =* 360) were sampled in all 17 villages, and production birds (*n =* 262) were actively being raised in 10 villages at the time of sampling. Environmental samples were collected from all 17 villages. The frequency and timing of sampling events for study sites are shown in Table S2 and Fig. S2 in the supplemental material. For all drugs, the custom breakpoint was lower than the official susceptible one, but for most drugs, the custom breakpoints were in the intermediate range as defined by CLSI. Table S3 shows the official resistant and susceptible breakpoints defined by CLSI, the custom breakpoint values estimated using our approach and subsequently applied in the analysis, and the percentage of isolates that would be classified as resistant under each scenario. Details of the fitted mixture models are available in Fig. S3.

**TABLE 1:** Counts of *E. coli* isolates collected from 17 villages in Esmeraldas Province, Ecuador, in 2010 to 2013 classified by sample type^a^

Sample type or age of bird	No. of *E. coli* isolates	No. of samples	No. of households	No. of villages
Poultry	1,875	622	226	17
Household	1,089	360	206	17
Production	786	262	35	10
<2 wks	297	105	10	6
3–5 wks	165	62	13	6
>6 wks	216	76	10	7
Data not available	108	37	13	5
				
Environment	1,460	529	190	17
Household water	326	144	114	17
Household soil	863	265	187	17
Coop soil	96	34	17	6
Household surfaces	98	54	46	8
Coop surface	77	32	14	5

a Production bird data include broilers and laying hens.

10.1128/mSphere.00021-15.3 Figure S2  Frequency of poultry and environment sampling by year and quarter. Download Figure S2, DOCX file, 0.02 MB. Copyright © 2016 Braykov et al. 2016 Braykov et al. This content is distributed under the terms of the Creative Commons Attribution 4.0 International license.

10.1128/mSphere.00021-15.4 Figure S3  Histograms of inhibition zones for production bird *E. coli* isolates and the fitted two-component mixture models. The proportion of isolates estimated to correspond to each of the two distributions is shown in the legend. We used expected maximum (EM) algorithms for repeated-measurement data to account for multiple isolates cultured from each sample. We compared parametric (parEM), semiparametric (spEM), and nonparametric (npEM) expectations. The best-fit model was selected based on the log-likelihood statistic, shown at the bottom of the graph. Larger zones of inhibition indicate that the isolate was less susceptible. Custom breakpoints were set where the density estimates for the fitted distributions intersect. That number is shown in blue. The black dotted line shows the official Clinical and Laboratory Standards Institute (CLSI) susceptibility breakpoint. Download Figure S3, DOCX file, 0.02 MB. Copyright © 2016 Braykov et al. 2016 Braykov et al. This content is distributed under the terms of the Creative Commons Attribution 4.0 International license.

### Antibiotic resistance in poultry samples. (i) Production versus household poultry.

**FIG 1:**
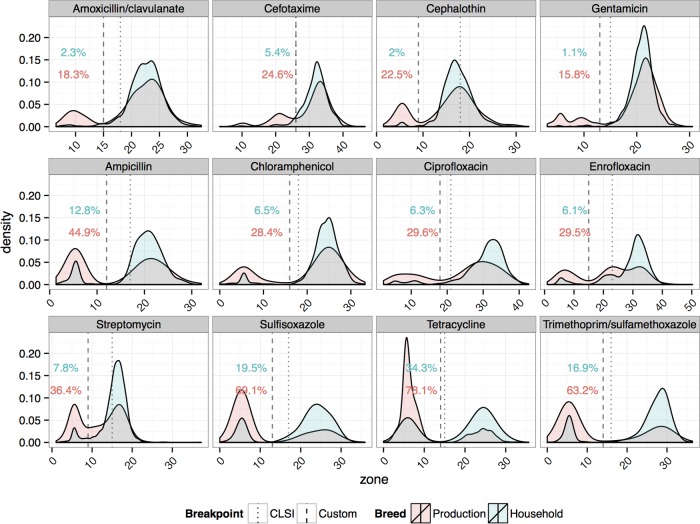
Kernel density estimates of inhibition zones and categorical interpretation of susceptibility tests of *E. coli* isolates. Distributions for samples from production birds (broilers and laying hens; *n =* 786 isolates from 262 birds) are shown in red, and distributions for household birds (*n =* 1,089 isolates from 360 birds) are shown in blue; overlapping portions are shown in gray. Percentages of resistant isolates in each sample are shown in corresponding colors. Larger zones of inhibition indicate that the isolate was less susceptible. Dashed lines show the custom susceptibility breakpoint that was derived from the use of a mixture model and was used to derive that categorical interpretation. Dotted lines show the consensus Clinical and Laboratory Standards Institute (CLSI) clinical breakpoints (46, 47).

Production birds, including broilers and laying hens, had high levels of resistance and a notably higher proportion of resistant isolates than household birds (resistance data were defined using our custom breakpoints). Resistance to tetracycline was detected in 78% of production birds and 34% of household birds. More than half of the production bird isolates were resistant to sulfisoxazole and trimethoprim-sulfamethoxazole (69% and 63%, respectively) compared to 20% and 17%, respectively, of the isolates from household birds (Table 2). The lowest resistance was to gentamicin (16% of production and 1% of household bird isolates) and amoxicillin/clavulanate (18% and 2%, respectively). The difference between production and household bird isolates was statistically significant (*P* < 0.01) for all drugs according to the results of analysis performed using generalized linear mixed models with a binomial identity link function (GLMM-logit) and was also reflected in the data showing significantly lower zones of inhibition (*P* < 0.01 for all drugs according to the results determined for continuous outcome measures using GLMM and analysis of variance [GLMM-ANOVA]) (Fig. 1).

**TABLE 2:** Percentages of *E. coli* isolates resistant to a panel of 12 antibiotics classified by sample type^a^

Sample type	% of *E. coli* isolates resistant to:											
	AMC	AM	CTX	CF	C	CIP	ENO	GM	S	G	TE	TMP
Poultry	9.01	26.24	13.44	10.61	15.68	16.11	15.89	7.25	19.79	40.27	52.64	36.32
Production (*n =* 786)	18.32	44.91	24.55	22.52	28.37	29.64	29.52	15.78	36.39	69.08	78.12	63.23
Household (*n =* 1,089)	2.30	12.76	5.42	2.02	6.52	6.34	6.06	1.10	7.81	19.47	34.25	16.90
												
Environment	6.44	18.97	6.03	5.41	7.05	4.52	4.59	3.01	6.71	23.29	31.30	20.89
Household water (*n =* 326)	12.27	26.38	4.60	10.12	6.13	4.29	4.60	2.15	4.60	26.07	29.14	23.01
Household soil (*n =* 863)	4.17	15.30	5.45	2.32	6.14	3.36	3.36	2.67	4.98	18.19	27.46	15.87
Coop soil (*n =* 96)	4.17	17.71	8.33	8.33	9.38	7.29	7.29	5.21	12.50	35.42	53.13	35.42
Household surfaces (*n =* 98)	2.04	20.41	3.06	2.04	6.12	1.02	1.02	0.00	9.18	24.49	28.57	19.39
Coop surfaces (*n =* 77)	15.58	28.57	19.48	20.78	19.48	19.48	19.48	11.69	24.68	51.95	59.74	51.95

a Numbers show percentages of isolates classified as resistant based on their zone of inhibition. Categorical interpretation is based on breakpoints derived as described in Materials and Methods. The number of isolates tested for each sample type is shown in Table 1. AMC, amoxicillin/clavulanate, AM, ampicillin; CTX, cefotaxime; CF, cephalothin; C, chloramphenicol; CIP, ciprofloxacin; ENO, enrofloxacin; GM, gentamicin; S, streptomycin; G, sulfisoxazole; TE, tetracycline; TMP, trimethoprim.

Examining the differences in the modality of zone distributions, we observed a phenotypic pattern of AR unique to production birds. Distributions for amoxicillin/clavulanate, cefotaxime, cephalothin, and gentamicin, shown in the top row of Fig. 1, showed bimodal tendencies for production birds, suggesting a mixed population of susceptible and resistant strains. Household birds had a unimodal distribution, with the exclusive presence of strains susceptible to these drugs. We refer here to this particular phenotypic pattern as a “production bird signature,” as this pattern suggests the presence of resistant phenotypes unique to production birds and not found in household birds. In contrast, distributions for all other drugs were bimodal for both types of poultry, suggesting that the same resistance phenotype is present in both samples, although the resistance phenotype was more prevalent in production birds for all drugs.

Resistance to at least one of the production signature drugs was present in 52.8% of production and 16% of household bird isolates during all sampling visits across all sites. Simultaneous resistance to the four drugs was found in 7.3% (57/786) of production bird isolates. While the phenotype was recovered in every quarter of the study period when poultry were sampled, 32 of the isolates were from one particular flock of chicks aged 10 to 14 days and purchased from a large city outside the immediate study area.

### (ii) Production birds by age.

**FIG 2:**
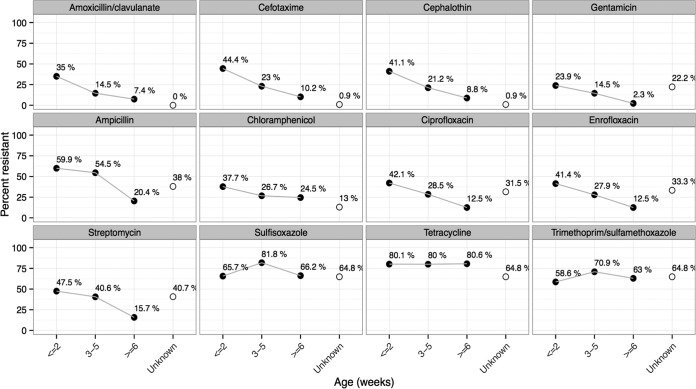
Categorical resistance of *E. coli* isolates from production birds (broilers and laying hens) classified by age of bird. Age data are based on results of surveys conducted at time of sample collection. *n =* 678 isolates from 225 birds had age available (*n =* 297 isolates from 105 birds aged ≤2 weeks; *n =* 165 isolates from 62 birds aged 3 to 5 weeks; *n =* 216 isolates from 76 birds aged ≥6 weeks). Black dots show the frequency of resistant isolates for each age group and transparent ones the frequency of resistant isolates for birds of unknown age (*n =* 108 isolates from 37 birds). Resistance categorization data are based on custom breakpoints. A generalized linear mixed-effects model of the zone of inhibition regressed against age, with bird category included as a random effect, showed a significant decline in resistance (*P* < 0.05) for all drugs with the exception of sulfisoxazole (*P* = 0.65), trimethoprim (*P* = 0.66), and tetracycline (*P* = 0.8).

The prevalence of resistant phenotypes tended to decrease with bird age (Fig. 2) for all drugs (*P* < 0.05 by GLMM-ANOVA) except those with the highest resistance levels (i.e., sulfisoxazole, trimethoprim-sulfamethoxazole, and tetracycline). This suggests that production birds are already colonized with strains resistant to some drugs when they are purchased and that this carriage declines with age. Age data were not available for household birds, but in comparisons of 105 birds of the youngest group (<2 weeks) to all 360 household ones, the production birds showed pronounced, characteristic differences in the modality of distributions (i.e., a “production signature”); in comparisons among 76 birds of the oldest group (>6 weeks), the modalities of the distributions did not differ (see Fig. S4A and B in the supplemental material). However, older production birds still retained resistance levels that were significantly higher than those determined for the household ones (*P* < 0.01 by GLMM-ANOVA and GLMM-logit).

10.1128/mSphere.00021-15.5 Figure S4  (A) Kernel density estimates of inhibition zones and categorical interpretation of susceptibility tests of *E. coli* isolates. Distributions for samples from production birds aged 2 weeks or less (broilers and laying hens; *n =* 297 isolates from 105 birds) are shown in red, and distributions for household birds (*n =* 1,089 isolates from 360 birds) are shown in blue; overlapping portions are shown in gray. Percentages of resistant isolates in each sample are shown in corresponding colors. Larger zones of inhibition indicate that the isolate was less susceptible. Dashed lines show the custom susceptibility breakpoint that was derived from the use of a mixture model and was used to derive that categorical interpretation. Dotted lines show the consensus Clinical and Laboratory Standards Institute (CLSI) clinical breakpoints (see references 6 and 7 in Supplemental Text S1). (B) Kernel density estimates of inhibition zones and categorical interpretation of susceptibility tests of *E. coli* isolates. Distributions for samples from production birds aged 6 weeks or more (broilers and laying hens; *n =* 216 isolates from 76 birds) are shown in red, and distributions for household birds (*n =* 1,089 isolates from 360 birds) are shown in blue; overlapping portions are shown in gray. Percentages of resistant isolates in each sample are shown in corresponding colors. Larger zones of inhibition indicate that the isolate was less susceptible. Dashed lines show the custom susceptibility breakpoint that was derived from the use of a mixture model and was used to derive that categorical interpretation. Dotted lines show the consensus Clinical and Laboratory Standards Institute (CLSI) clinical breakpoints (see references 6 and 7 in Supplemental Text S1). Download Figure S4, DOCX file, 0.2 MB. Copyright © 2016 Braykov et al. 2016 Braykov et al. This content is distributed under the terms of the Creative Commons Attribution 4.0 International license.

10.1128/mSphere.00021-15.5 Text S1  Additional experimental details. Download Text S1, DOCX file, 0.02 MB. Copyright © 2016 Braykov et al. 2016 Braykov et al. This content is distributed under the terms of the Creative Commons Attribution 4.0 International license.

### (iii) Production birds by point of purchase.

**FIG 3:**
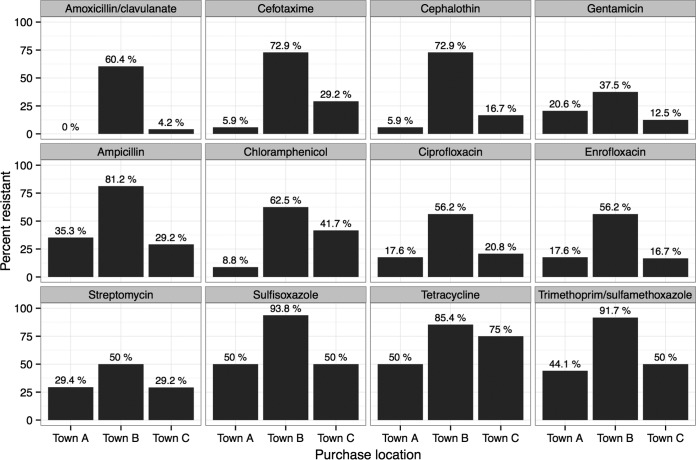
Antibiotic resistance in *E. coli* isolates from production birds (broilers and laying hens) of age <2 weeks by purchase location. Locations are based on results of surveys conducted at the time of sample collection. Resistance categorization data are based on custom breakpoints. The data from town A (*n =* 34 isolates from 12 samples) and town C (*n =* 24 isolates from 9 samples) were significantly different (*P* < 0.05) from the data from town B (*n* = 48 isolates from 17 samples) by GLMM-ANOVA and GLMM-logit. *n =* 106 isolates from 38 samples.

Information on the purchase source of flocks was available for 38 of 105 production birds of age <2 weeks. The prevalence of AR among these samples differed by the location where chicks were purchased (Fig. 3). This trend was significant for 11 of the 12 drugs (*P* < 0.01 by GLMM-logit). Resistance, particularly to the production bird signature drugs (shown in the top row of Fig. 3), was always highest for chicks purchased in town B, a large city outside the immediate study area. Resistance in broilers from towns B and C decreased with age (see Fig. S5 in the supplemental material). For town A, the direction of the change depended on the initial starting levels. Irrespective of source, the trend was for levels of resistance in older birds to converge toward those shown by domestic birds. Data forms used during sampling recorded only the town from which the birds were sourced and not the specific stores. However, the field workers noted no more than 5 stores, and usually 1 to 3, in each of the 3 towns. Our ethnographic interviews with a veterinary store owner in town B revealed that he regularly supplemented the chicks’ drinking water with antibiotics as soon as they arrived at his shop and before resale to customers. The variation in resistance patterns could be at the level of the town or, more likely, at that of a particular veterinary store.

10.1128/mSphere.00021-15.6 Figure S5  Antibiotic resistance in *E. coli* isolates from production birds (broilers and laying hens) by age and purchase location. Location and age data are based on results of surveys conducted at time of sample collection. Resistance categorization data are based on custom breakpoints. *n =* 156 isolates from 45 samples in town A, *n* = 98 isolates from 36 samples in town B, and *n* = 39 isolates from 16 samples in town C. Download Figure S5, DOCX file, 0.4 MB. Copyright © 2016 Braykov et al. 2016 Braykov et al. This content is distributed under the terms of the Creative Commons Attribution 4.0 International license.

### (iv) Production birds with added antibiotics in water.

Detailed surveys on poultry rearing practices were available for 96 production birds in 20 households in nine villages. Farmers reported administering penicillin plus streptomycin, tetracycline, sulfonamide, sulfamethazine plus trimethoprim, piperacillin, erythromycin, sulbactam, and/or enrofloxacin. Of 20 farming households surveyed in detail about poultry-raising practices, 16 (80%) reported supplementation with antibiotics beyond what is already available in feed. No significant differences were observed between birds with and without reported supplemental antibiotic administration (*P* > 0.05 for all drugs by GLMM-ANOVA and GLMM-logit).

### Antibiotic resistance in environmental samples. (i) Surface isolates from coops and households.

**FIG 4:**
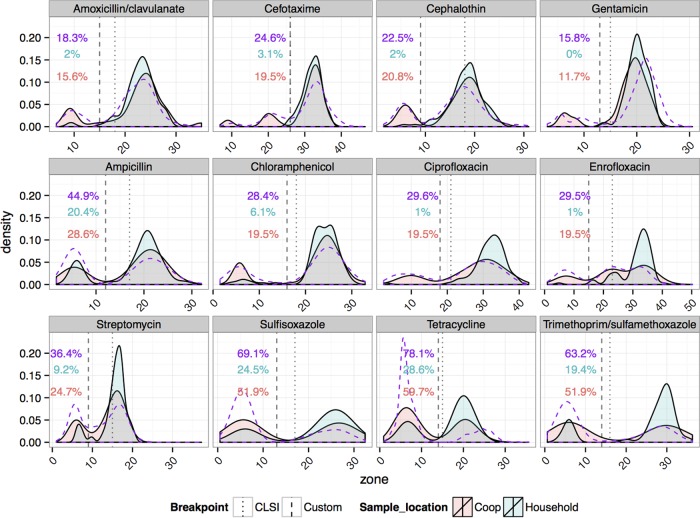
Kernel density estimates of inhibition zone profiles and categorical interpretation of susceptibility tests for *E. coli* isolates from household and coop surfaces and from production birds (broilers and laying hens). Kernel density estimates for the distributions of isolates from coop (red) and household (blue) and production bird (dashed purple) samples are shown; overlapping coop and household portions are colored in gray. Percentages of resistant isolates in each sample are shown in corresponding colors. Dashed vertical lines show the custom susceptibility breakpoint that was derived from the use of a mixture model and was used to derive that categorical interpretation. Larger zones of inhibition indicate that the isolate was less susceptible. Dotted lines show the consensus clinical breakpoints used by the Clinical and Laboratory Standards Institute (CLSI) (46, 47). For coops, *n =* 77 isolates from 32 samples; for households, *n =* 90 isolates from 54 samples; for production birds, *n =* 786 isolates from 262 birds.

Resistance patterns in isolates from birds in coops were similar to the patterns in those from production birds but different from the patterns in household isolates. Surface isolates from coops showed higher resistance levels than surface isolates from the domestic environment (*P* < 0.05 by GLMM-logit and GLMM-ANOVA, with the exception of ampicillin and chloramphenicol) (Fig. 4). In addition, surface isolates from coops exhibited the production bird signature pattern of resistance (shown in the top row of Fig. 4). Simultaneous resistance to the four signature drugs was detected in 5 of 72 recovered isolates (6.5%), all from the same site where the flock of chicks with high prevalence of the same strain was sampled.

### (ii) Water and soil isolates by farming history of household.

Using household survey data on past experiences with production poultry farming in all villages, we divided households into those that had farmed production birds within the previous year, those that had farmed production birds over 1 year before the sampling event, and those that had never farmed production birds. We saw no differences in inhibition zone profiles, and their categorical interpretations showed no significant differences across groups for household water samples and only minor differences across groups for household soil samples (see Fig. S6A and B in the supplemental material).

10.1128/mSphere.00021-15.7 Figure S6  (A) Kernel density estimates of inhibition zone profiles and categorical interpretation of susceptibility tests for *E. coli* isolates from household drinking water samples classified by farming history of household. Based on survey data, households were divided into those that had farmed broiler chickens or laying hens (“production birds”) within the previous year (*n =* 82 isolates from 38 samples), those that had farmed production birds over 1 year before the sampling event (*n =* 61 isolates from 28 samples), and those that had never farmed production birds (*n =* 188 isolates from 76 samples). Larger zones of inhibition indicate that the isolate was less susceptible. Colored numbers show the percentages of resistant isolates according to the custom susceptibility breakpoint, where the color corresponds to the exposure. There were no significant differences between groups (*P* > 0.05 by GLMM-ANOVA and GLMM-logit). (B) Kernel density estimates of inhibition zone profiles and categorical interpretation of susceptibility tests for *E. coli* isolates from household soil samples classified by farming history of household. Based on survey data, households were divided into those that had farmed broiler chickens or laying hens (“production birds”) within the previous year (*n =* 225 isolates from 71 samples), those that had farmed production birds over 1 year before the sampling event (*n =* 185 isolates from 58 samples), and those that had never farmed production birds (*n =* 451 isolates from 131 samples). Larger zones of inhibition indicate that the isolate was less susceptible. Colored numbers show the percentages of resistant isolates according to the custom susceptibility breakpoint, where the color corresponds to the exposure. There were significant differences between groups for amoxicillin/clavulanate (*P* < 0.01 by GLMM-ANOVA), ampicillin (*P* = 0.04), cephalothin (*P* < 0.01), sulfisoxazole (*P* < 0.01), and trimethoprim (0.03). Download Figure S6, DOCX file, 0.03 MB. Copyright © 2016 Braykov et al. 2016 Braykov et al. This content is distributed under the terms of the Creative Commons Attribution 4.0 International license.

### (iii) Water and soil isolates by farming history of village.

We were also interested in whether poultry farming had an effect on AR profiles at a larger scale. Using the monthly survey data on poultry production, villages were categorized into high-, medium-, and low-intensity farming sites, respectively defined as >400 broilers, 100 to 400 broilers, and <100 broilers raised in the year prior to environmental sampling. Again, we saw only minor differences in inhibition zone profiles and their categorical interpretations across these village groups for household water and soil samples (see Fig. S7A and B in the supplemental material).

10.1128/mSphere.00021-15.8 Figure S7  (A) Kernel density estimates of inhibition zone profiles and categorical interpretation of susceptibility tests for *E. coli* isolates from household drinking water samples classified by intensity of farming in the year prior to sampling. Villages were classified as high-intensity (>400 birds; *n =* 113 isolates from 55 samples), medium-intensity (100 to 400 birds; *n =* 40 isolates from 19 samples), and low-intensity (<100 birds; *n =* 173 isolates from 70 samples) sites based on the maximum recorded number of birds in the year preceding the sampling visit. Larger zones of inhibition indicate that the isolate was less susceptible. There were no significant differences between groups, with the exception of the ampicillin (P = 0. 008 by GLMM-logit) and sulfisoxazole (*P* = 0.01) data, in which cases samples from villages without poultry farming in the previous year had higher resistance. (B) Kernel density estimates of inhibition zone profiles and categorical interpretation of susceptibility tests for *E. coli* isolates from household soil samples classified by intensity of farming in the year prior to sampling. Villages were characterized as high-intensity (>400 birds; *n =* 279 isolates from 90 samples), medium-intensity (100 to 400 birds; *n =* 135 isolates from 44 samples), and low-intensity (<100 birds; *n =* 449 isolates from 131 samples) sites based on the maximum recorded number of birds in the year preceding the sampling visit. Larger zones of inhibition indicate that the isolate was less susceptible. There were no significant differences between groups, with the exception of the amoxicillin/clavulanate (*P* = 0.03 by GLMM-ANOVA), gentamicin (*P* < 0.01 by GLMM-logit), and tetracycline (*P* < 0.01 by GLMM-ANOVA) data, in which cases samples from villages with medium-intensity poultry farming in the previous year tended to be more resistant than those from high- or low-intensity villages. Download Figure S7, DOCX file, 0.6 MB. Copyright © 2016 Braykov et al. 2016 Braykov et al. This content is distributed under the terms of the Creative Commons Attribution 4.0 International license.

### Cluster analysis of resistance patterns across all sample sources.

**FIG 5:**
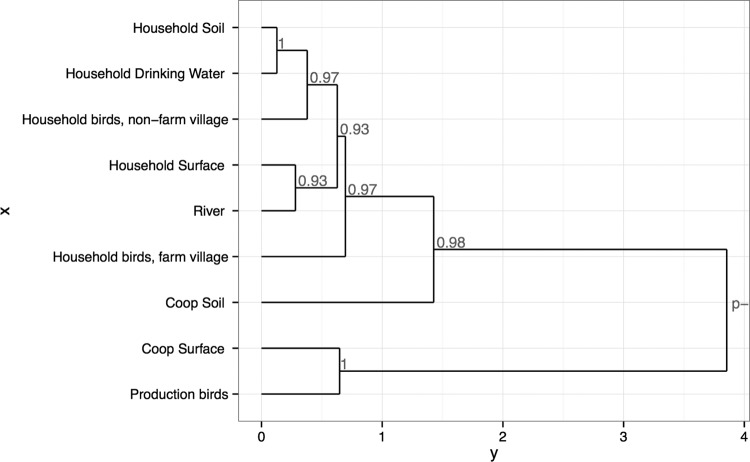
Hierarchical clustering by sample type and location. Data are based on resistance profiles of 3,860 *E. coli* isolates of environmental and poultry samples. Numbers show *P* values from multiscale bootstrap resampling performed with *n* = 1,000 replications.

To visualize the relative similarities of AR patterns across all sample types, we performed hierarchical clustering analysis using the normalized zones of inhibition. A dendrogram, which includes bootstrap support values (Fig. 5), shows that production bird and coop surface isolates form a clade distinct from that seen with household birds, water, and soil isolates. Coop soils and cloacal isolates from household birds raised in villages with active farming are closest to the production bird phenotypic pattern.

## DISCUSSION

This study used a multidisciplinary approach to examine populations of *E. coli* circulating in the production versus domestic environment in the context of small-scale poultry farming in rural Ecuador. We observed high levels of AR in both the production and household contexts, although the isolates from production-environment-associated samples showed substantially higher levels of resistance than isolates from domestic-environment-associated samples. Production birds exhibited a phenotypic pattern distinct from that seen with household birds, and this signature pattern was also prevalent in poultry coops but not in domestic environments. Levels of AR were higher among younger birds and were associated with a particular source of chicks; both findings suggest introduction from outside sources. Poultry farming status was not associated with AR in isolates from domestic environments in comparisons of farming to nonfarming households or across villages with different farming intensity levels. These results suggest that AR strains associated with poultry production likely originate from sources outside the study area and that these outside sources might be a better target for control efforts than local management practices.

### Phenotypic resistance in production environments.

In the *E. coli* isolates that we recovered from production birds, we found high levels of resistance to drugs used in human medicine, in particular, tetracycline, trimethoprim-sulfamethoxazole, and sulfamethoxazole, to which over half of the isolates were resistant. Rates of resistance to broad-spectrum antibiotics amoxicillin/clavulanate (23% by CLSI breakpoint and 18.3% by custom breakpoint) and ciprofloxacin (32% and 30%, respectively) were substantially higher than those found via abattoir surveillance carried out on industrial farms in the United States (9% and <1%, respectively) (22) and in Canada (8% resistance to nalidixic acid) (23). We found relatively high levels of resistance to broad-spectrum antibiotics even in the household environment, particularly in drinking water, where the proportion of isolates resistant to amoxicillin/clavulanate, ampicillin, and cephalothin, drugs used for human medicine, was higher than in household birds. Surprisingly, resistance to chloramphenicol was uncommon, although our chemical analysis showed it to be an additive to commercial feed.

In line with studies from other regions in the world, AR in intensively farmed animals tends to be higher than in free-range or organically raised varieties (24–27). For example, a study of 20 poultry flocks with over 500 birds from Germany found that resistance rates and mean MICs of bacteria isolated from organic keeping systems had lower values than those from conventional ones, especially for *E. coli*. *E. coli* and enterococcal isolates from free-range Tibetan pigs (*n =* 232) had lower levels of resistance, particularly to tetracycline and other antibiotics known to be used in farming, than those from intensively raised pigs in other parts of China (28).

Resistance to amoxicillin/clavulanate, cefotaxime, cephalothin, and gentamicin was specific to production bird isolates. Because AR is passed from the chickens to the immediate production environment, poultry production may serve as a source of occupational exposure to AR strains of bacteria. Results of a genotypic analysis of human isolates from the same study system (K. A. Moser, L. Zhang, I. Spicknall, N. Braykov, K. Levy, C. F. Marrs, B. Foxman, G. Trueba, W. Cevallos, J. Trostle, and J. N. S. Eisenberg, unpublished data) point to a molecular basis for these phenotypic groupings. Isolates from human chicken farmers had an elevated prevalence of the *int1* gene platform, which was associated with increased resistance to amoxicillin-clavulanate, cephalotin, cefotaxime, and gentamicin; isolates resistant to these drugs were almost exclusively found in production birds (29).

### Extrinsic sources of resistance.

We did not detect differences between birds reportedly receiving antibiotic supplements in the diet, in addition to what is already present in commercially mixed feed. While this counters the results of other studies (30, 31), the exposure in our data was based on self-reporting and is therefore subject to recall and/or misclassification bias. We find more evidence in support of the alternative explanation that production chickens are colonized with resistant strains in the hatcheries prior to their arrival in village coops.

The decline of resistance with production bird age suggests that farmed poultry start their growth cycle precolonized with antibiotic-resistant bacteria rather than acquiring resistance in the village environment as a result of any particular farming practice. This pattern has been previously reported in the literature. For example, a study of 293 *E. coli* isolates collected from French laying hens reported higher carriage in younger birds of *bla_CTX-M_* genes that confer resistance to most beta-lactam antibiotics (32). A large case-control study in Canada of 197 isolates from broilers sampled longitudinally and exposed to different antibiotic regimens also found an overall decrease in the prevalence of resistance between days 7 and 35 in both cases and controls and a concurrent decrease in carriage of the *int1* integron and *tet* gene (30). One possible explanation for age-dependent AR is that there is a dilution effect: the selection pressure of a fixed dose of antimicrobial becomes weaker as animals increase in mass. Another is that AR strains are “age adapted” to the physiology of younger hosts: an experimental study of neonatal calves supports this hypothesis, as animals inoculated with susceptible and resistant *E. coli* strains shed significantly greater concentrations of the latter, suggesting a fitness advantage for AR strains (33).

While the overall decline in resistance prevalence appears to be part of the normal course of *E. coli* community dynamics or changes in the host’s physiology, we also found that certain phenotypes such as the production bird signature are imported from outside the study villages. This finding is supported by the data indicating that chicks purchased from one particular town, a large regional center where the owner of one of the town’s few veterinary stores reported heavily supplementing the chicks with antibiotics, were several times more likely to be resistant to all drugs, especially the ones with the production bird signature.

The potential of young animals to act as sources of AR suggests that local management practices should focus on poultry hatcheries and sources along the distribution chain to control the spread of AR associated with small-scale poultry farming. Treatment of breeding hens with antibiotics may pass AR bacteria vertically from the hen to the egg and chick, even if no antibiotics are administered *in ovo* and/or to the chicks.

### Risks of exposure to poultry farms.

We found that samples from surfaces of poultry coops had resistance profiles most similar to those of samples from production birds. The high rates of AR that we observed on the surfaces of poultry production facilities in these villages may present a more localized occupational risk for acquisition of AR by poultry farmers and have the potential to impact humans living in the communities through contact with farmers as well as poultry consumption. In a related study in the villages under study here, we found that human fecal samples from poultry farmers exhibited higher levels of phenotypic and genotypic (class-1 integron) resistance, with the prevalence of *int1* among farmers of production birds over twice as high as the prevalence among those who raised household ones (Moser et al., unpublished). This suggests that occupational exposure to poultry farming is associated with the carriage of more-resistant strains, similarly to what has been found in larger-scale industrial operations (15, 24, 34). The idea that farming is an occupational risk for AR is supported by literature dating back to the 1970s (14; see also a review in reference 35). Our findings with respect to AR and coop surfaces suggest that direct handling of birds is a potential route of transmission.

While we did observe the production signature for AR in the immediate production environment, this signature production pattern of AR did not occur in the domestic environment, suggesting that AR selected for by poultry production systems is limited to the production environment. It is possible that the frequency of sampling and the culturing methods that we used did not offer enough power to detect the effect of added systemic antibiotics that other studies have reported (24, 30, 36, 37). However, it is also possible that the intermittent nature of intensive production does not result in sufficient pressure to affect bacterial populations within the household environment. The variability of intensity of poultry farming across space and time has implications for the spread of AR in this region and likely plays a role in limiting the impact of farming to the immediate production environment. Many of the drivers of the “boom and bust” nature of the enterprise that we identified through ethnography, such as access to capital and seasonality in markets as barriers to reinvestment, are likely at work in other developing regions as well.

Our finding that chloramphenicol is present in commercial poultry is disconcerting. The use of the drug in human medicine has been limited due to concerns over development of aplastic anemia, a potentially fatal bone marrow disorder. Chloramphenicol use in food animal is not approved in developed countries as studies have shown residues in meat in concentrations sufficient to suppress bone marrow development (38).

### Sensitivity of results to custom breakpoints.

This study utilized unique interdisciplinary methods to provide insights into the ecology of AR at the interface of humans and animals. Unlike most environmental studies that summarize AR using clinical antibiogram methods, we used the full phenotypic resistance profile (i.e., the full distribution of zones of inhibition), which is a cost-effective way to study the epidemiology of drug-resistant strains in the absence of molecular data (39, 40). Analyzing the distribution of zone diameters in addition to categorical interpretations allowed us to identify a signature phenotypic pattern unique to production birds that suggested that AR associated with poultry production likely originates outside the studied communities.

We used modeling techniques to classify isolates into resistant and susceptible bacterial populations after observing from our empirical data that the preestablished breakpoints did not accurately reflect the distributions of zones of inhibition in the bacterial populations in our study. Our approach is not suitable for classifying clinical isolates, as it disregards pharmacokinetic and pharmacodynamics properties of antibiotics, but was better suited to ecological studies because of its ability to empirically distinguish between the populations of bacteria in our samples. While our major conclusions were not sensitive to the use of custom susceptibility criteria, we show that the use of clinical breakpoints may misclassify over 30% of isolates for some antibiotics (amoxicillin/clavulanate, cephalothin, streptomycin, and enrofloxacin), potentially leading to problems in the interpretation of results (see Table S3 in the supplemental material). In these cases, use of the CLSI breakpoints would have resulted in classifying as resistant the isolates that were more likely to belong to the susceptible population. This point is well illustrated by comparing the results for enrofloxacin and ciprofloxacin: the zones of inhibition for these antibiotics, both fluoroquinolones, had a Person correlation of 0.951, and one would therefore expect the proportions of isolates resistant to the two antibiotics to be similar. The use of CLSI breakpoints would have classified 24% of the isolates as enrofloxacin resistant and 12.4% as ciprofloxacin resistant, while our custom breakpoints yielded frequencies of 10.9% and 11%, respectively.

### Study limitations and avenues for future research.

This study was limited by several factors. First, because of the nature of our visits to the villages, we were unable to exploit the wide variability in animal densities over time to look more closely at the correlation between temporal variability and resistance patterns. Second, our analysis relies on phenotypic resistance data, and we therefore did not have the opportunity to account for the multiple genetic determinants and expression patterns that underlie resistance. Third, we did not test for resistance to two of the three antibiotics that our chemical analysis found in the poultry feed—virginiamycin and lincomycin, both narrow-spectrum antibiotics active against Gram-positive bacteria and not against *E. coli*. Fourth, we used *E. coli* as a sentinel organism and relied on culture-based methods, capturing only a fraction of the complex, multilevel interactions between environment, host, microorganism, and horizontally transferred genetic elements. Future similar studies could use a metagenomic approach to characterize the diversity of environmental and animal reservoirs as well as the patterns and mechanisms of resistance gene exchange between these bacterial communities (7).

Despite these limitations, our report provides a multidisciplinary, ecological framework and a large data set to characterize the prevalence of resistance among animals and household environments in the context of small-scale farming. This type of analysis is needed to understand the implications of the expansion of small-scale poultry farming in developing countries currently promoted as an economic development strategy.

## MATERIALS AND METHODS

### Study area.

The study was conducted between August 2010 and July 2013 in a rural region of Esmeraldas Province, in northern coastal Ecuador. Community members primarily consume untreated surface source water, and sanitation facilities are inadequate (41). Our research team has been working in a total of 31 communities in this region since 2003; the study sites and region are described in detail elsewhere (21, 42). The present study was conducted in a subset of 17 villages where we were able to collect environmental and animal samples.

### Ethnography.

To understand the sociocultural context of the sampling and biological analysis, ethnographers conducted structured interviews with local stakeholders. Information was gathered on the practices, organization, economics, and history of poultry farming, including antibiotic use and the rearing and consumption of different kinds of birds.

### Household surveys.

We visited villages monthly to record the total number of production birds in each community. In addition, we conducted a detailed survey within 10 days of sample collection on the type, size, age, origin, and intended use of flocks, the brands and types of feed used, and the use of supplementation with antibiotics.

Informed consent was obtained from all participating households. The Institutional Review Boards of the University of Michigan, Universidad San Francisco de Quito, Trinity College, and Emory University approved all interactions with human subjects.

### Sample collection and laboratory analysis.

In villages with production poultry farming, teams visited (i) all households with active poultry coops and (ii) an equivalent number of nonfarming households that were located far from coops. If villages had no poultry farming at the time of the visit, 3 to 10 nonfarming households were chosen at random, depending on the size of the village. Additional details on study design, populations, and sample collection methods are available in the supplemental material.

### Poultry samples.

The communities were visited 1 to 4 times between August 2010 and November 2013 to sample “production birds” and “household birds.” Production birds included breeds of laying hens raised for egg production or broiler chickens that are raised in coops for 6 to 7 weeks before slaughter. These birds eat formulated feed containing antibiotics and are also commonly given antibiotics as prophylaxis via water. Capacities of coops ranged from ~50 to ~100 birds of a single age for a typical single household coop to ~1,000 birds of multiple ages for the group facility. Thirteen households in seven villages were engaged in rearing production birds. In each coop, we sampled five production birds of each age group (categorized in weeks of age).

Household birds included varietals not intended for commercial sale that are not held in coops, that eat scraps and ground maize rather than formula feed, and that do not receive antibiotics. We sampled 5 to 10 household birds from each village, regardless of whether the villagers were actively engaged in production poultry farming.

For all birds, we collected cloacal samples using sterile swabs that were placed in Cary Blair transport medium (Becton, Dickinson, Franklin Lakes, NJ) and streaked directly on MacConkey lactose (MKL) agar for isolation.

### Environmental samples.

Samples from household drinking water, soil from house surroundings, and kitchen surfaces were collected from households associated with production or household birds in order to characterize the domestic environment. In addition, soil and surface samples were collected from coops in order to characterize the production environment.

### Household water.

During the first half of the study period, between August 2010 and January 2012, we collected 50-ml water samples from two different household storage containers in Whirl-Pak bags (Nasco Corp., Fort Atkinson, WI) in the same manner that water was dispensed for drinking. If there was only one drinking water container available, then the second sample was taken from water used to wash dishes or bathe. For the remainder of the study period, only one sample per household was obtained from drinking containers in order to reduce sampling effort, as there were no notable differences in the *E. coli* isolates recovered across repeated samples. Samples were processed using membrane filtration for isolation of *E. coli*.

### Soil.

During the first half of the study period, we collected two samples of approximately 15 cm^3^ from around the house and from around the poultry coop, if one was present. Soil from just below the surface was placed into a conical tube using a sterile plastic spoon that was discarded after use. Samples were stored on ice until processing in the laboratory was performed (within 4 to 6 h). For the second part of the study period, only a single sample was obtained from the household yard, due to the rarity of coops and difficulty in recovering *E. coli* from these samples.

### Surfaces.

During the first half of the study period, we collected household surface samples from two locations, namely, where food was prepared (e.g., a cutting board) and where food was eaten (e.g., a table). A 28-by-30-cm plastic stencil was used to define a consistent sampling area. Two surface samples from the inside or outside the coop, which was usually constructed from cement or wood, were also taken using the same procedures. Surface samples were plated directly onto Chromocult agar and streaked for isolation. For the second part of the study period, surface samples were not collected due to the low rate of isolate recovery.

### Sample processing.

Samples were first plated on Chromocult agar, and then presumptive *E. coli* colonies were transferred onto MacConkey lactose (MKL) agar for confirmation before replating on Chromocult was performed to ensure pure isolates. We selected up to four *E. coli* colonies from soil and fecal samples and two from water and surface samples to test for AR. More isolates from soil samples than from water or surfaces were used because we expected higher microbial diversity in soil. Antibiotic sensitivity was assessed using the Kirby-Bauer disc diffusion method (43) and 12 antibiotics: ampicillin, amoxicillin/clavulanate, cefotaxime, cephalothin, chloramphenicol, ciprofloxacin, enrofloxacin, gentamicin, streptomycin, sulfisoxazole, trimethoprim-sulfamethoxazole, and tetracycline (Becton, Dickinson, Franklin Lakes, NJ). Zones of inhibition were measured after a 24-h incubation period using digital calipers.

### Statistical analysis.

Our main outcome of interest was the zone of inhibition around each of the tested antimicrobial discs. A secondary outcome was the categorical interpretation of the zone (susceptible or nonsusceptible), based on custom breakpoints that we defined based on zone size distributions.

Most studies of AR construct antibiograms using categorical interpretations (susceptible, resistant) of MICs or corresponding disc diffusion zones of inhibition based on externally defined consensus breakpoints set by organizations such as the U.S. Clinical and Laboratory Standards Institute (CLSI) or its European equivalent (EUCAST). However, these breakpoints have limited utility in distinguishing between distinct populations of organisms in circulation. Because the primary objective of our study was to understand the ecological dynamics of AR *E. coli* within production and domestic environments in our study villages, we took advantage of the full data on the distributions of zones of inhibition of each antibiotic for all isolates in order to gain additional insights into the populations of *E. coli* in the different environments that we sampled.

We used differences in the modalities of zone distributions to identify populations of isolates with distinct phenotypic resistance patterns. Where we classified isolates as resistant or susceptible, we used empirically derived custom susceptibility breakpoints, because examination of the distributions of the zones of inhibition showed that the externally defined breakpoints based on wild-type cutoff values (44) did not accurately reflect the local population of *E. coli* strains. These custom breakpoints have limited clinical utility, but they improved our ability to distinguish between populations of bacteria in our sample. More details on the analytical methods to define susceptibility breakpoints are available in the supplemental material.

Distributions suggested a population of all resistant or all susceptible bacteria when Hartigans’ dip statistic (which measures multimodality in a sample) had a *P* value of >0.1, failing to reject the null hypothesis of unimodality, or when the estimated proportion of a component in a mixture was lower than 0.01. Otherwise, the distributions were considered bimodal, suggesting a mixture of susceptible and resistant bacterial populations.

We compared the kernel density estimates of zone distributions and the percentages of nonsusceptible isolates across categories of interest. We used generalized linear mixed models (GLMM) to model the outcome of AR. An identity link function was used for continuous outcome measures (inhibition zone size; GLMM-ANOVA) and a binomial one for the categorical interpretation of the zones (susceptible and nonsusceptible; GLMM-logit). In all specifications, we used nested random effects with various intercepts and slopes for analysis at the sample, household, and village levels to capture unobserved heterogeneity between the multiple levels and to account for repeated sampling.

The resistance profile for each isolate was also used to perform hierarchical cluster analysis. We normalized the zones of inhibition, computed Euclidean distances between isolates, and ran a hierarchical clustering algorithm with an agglomeration method based on the average distance between categories, implemented in R’s flashClust package (45). Support values were calculated via multiscale bootstrap resampling with *n* = 1,000 replications.

All analyses were carried out in R version 3.0.1 (R Foundation for Statistical Computing, Vienna, Austria).

### Poultry feed samples.

Prior to the start of the study, we sampled seven varieties of the most commonly sold brand of feed (Nutril) from a veterinary store in the central trading city of Borbón in July 2009. Every type of feed sold was sampled, including starter, fattening, and finishing feed for broilers and layer hens. Samples were tested for the presence of the following antibiotics using mass spectrometry techniques: lincomycin, virginiamycin, bacitracin, flavomycin, avilomycin, tylosin, nitrofurantoin, chloramphenicol, tetracycline, sulfamethazine, and sulfathiazole. With the exception of nitrofurantoin, the first eight drugs in the panel belong to antibiotic classes that are exclusively used to treat and prevent infections with Gram-positive bacteria and therefore were not included in susceptibility testing of *E. coli* isolates (see Table S1 in the supplemental material for details).

10.1128/mSphere.00021-15.9 Table S1  Chemical analysis of feed samples of the most commonly sold brand of feed (Nutril) purchased at a veterinary store in the large regional center. Samples were tested for the presence of lincomycin, virginiamycin, bacitracin, flavomycin, avilomycin, nitrofurantoin, chloramphenicol, tetracycline, tylosin, sulfamethazine, and sulfathiazole by mass spectrometry. Chemical extractions were carried out at Emory University laboratories in April 2010 using standard protocols. High-performance liquid chromatography–high-resolution tandem mass spectrometry using an Orbitrap (ThermoFinnigan, San Jose, CA) was carried out in laboratories at the U.S. Centers for Disease Control and Prevention. Information on the concentration of antibiotics in collected samples was not available. Download Table S1, DOCX file, 0.5 MB. Copyright © 2016 Braykov et al. 2016 Braykov et al. This content is distributed under the terms of the Creative Commons Attribution 4.0 International license.

10.1128/mSphere.00021-15.10 Table S2  Numbers of samples and recovered isolates classified by site, poultry farming intensity, and month and year of sampling visit. Villages were classified into groups based on the maximum recorded number of birds during monthly census surveys (see Fig. S1). Two sites (sites A and N) did not have such census data available. Download Table S2, DOCX file, 0.01 MB. Copyright © 2016 Braykov et al. 2016 Braykov et al. This content is distributed under the terms of the Creative Commons Attribution 4.0 International license.

10.1128/mSphere.00021-15.11 Table S3  Official and custom susceptibility breakpoints. Custom breakpoints (BPs) were used to derive categorical resistance profiles for all 3,860 isolates used in the analysis. CLSI, Clinical and Laboratory Standards Institute. Custom breakpoints were defined using two-component mixture models for zone distributions and parametric, semiparametric, and nonparametric expectation maximum algorithms for repeated measurement data to account for multiple isolates cultured from each sample. The best-fit model was selected based on the log-likelihood statistic. Custom breakpoints were set where the density estimates for the fitted distributions intersect. These updated breakpoints were rounded to the nearest whole number and used to categorize all isolates in our data as susceptible or resistant. CLSI breakpoints were assigned on the basis of accepted veterinary breakpoints (6) or of clinical ones for drugs not approved for veterinary use (7). Download Table S3, DOCX file, 0.02 MB. Copyright © 2016 Braykov et al. 2016 Braykov et al. This content is distributed under the terms of the Creative Commons Attribution 4.0 International license.
