# Supplementary material for: Antibiotic Resistance in Animal and Environmental Samples Associated with Small-Scale Poultry Farming in Northwestern Ecuador
Source: mSphere. 2016 Feb 10;1(1):e00021-15. doi: 10.1128/mSphere.00021-15 (PMC4863614; doi:10.1128/mSphere.00021-15)
Supplement: Text S1 [file sph001162003s1.docx]

**SUPPLEMENTAL MATERIAL**

***Sampling methods***

*Poultry*

For all birds, we collected cloacal samples using sterile swabs that were placed in Cary Blair transport medium (Becton Dickinson, Franklin Lakes, NJ ) and streaked directly on McConkey-Lactose agar for isolation.

*Household water*

Samples were processed using membrane filtration for isolation of *E. coli*. Two volumes varying between 3-50mL of water were filtered depending on the expected concentrations of *E. coli* for a particular water source, based on prior field testing. Because the goal was to recover and isolate *E. coli*, it was not necessary to keep the quantities uniform. Membranes were plated onto Chromocult agar (Merck, Darmstadt, Germany) and incubated at ambient temperature for 48 hours.

*Soil*

Each soil sample was diluted with 30mL of deionized water and mixed thoroughly to obtain a 2:1 dilution. A second dilution was made with an additional 10mL of deionized water. The supernatant of the original sample and the dilution were streaked separately onto Chromocult agar for isolation.

*Surface*

A 28x30cm plastic stencil was used to define a consistent sampling area. If the area was dry, we used a cotton tip applicator that had been submerged in 1mL 0.9% NaCl solution to obtain the sample. If the area was wet, a dry swab was used and placed in Cary Blair transport medium (Becton Dickinson, Franklin Lakes, NJ). Surface samples were plated directly onto Chromocult agar and streaked for isolation.

*Sample processing*

All plates were incubated at ambient temperature, ranging from 24-30^o^C. After approximately 24-48 hours, up to four 4-methylumbelliferyl-beta-D-glucuronide (MUG) -positive *E. coli* colonies from each sample isolated on Chromocult agar were randomly selected and transferred onto MacConkey Lactose (MKL) agar to confirm the presence of *E.* *coli*. If *E. coli* was not initially isolated upon first plating on Chromocult agar due to its low abundance or contamination with other bacteria, efforts were made to re-isolate colonies on Chromocult agar before transferring onto MKL agar. After a 24-hour incubation at 37^o^C, lactose-positive (lac+) *E. coli* colonies were transferred from MKL back onto Chromocult to ensure pure isolates, and incubated for another 24 hours.

***Custom susceptibility breakpoints***

Most studies of AR construct antibiograms using categorical interpretations (susceptible, resistant) of minimum inhibitory concentrations or corresponding disc diffusion zone of inhibition based on externally defined consensus breakpoints set by organizations such as the US Clinical Laboratory Standards Institute (CLSI) or its European equivalent EUCAST. However, these breakpoints have limited utility in distinguishing between distinct populations of organisms in circulation. Because the primary objective of our study was to understand the ecological dynamics of AR *E. coli* within production and domestic environments in our study villages, we took advantage of the full data on the distributions of zones of inhibition of each antibiotic for all isolates, in order to gain additional insights into the populations of *E. coli* in the different environments we sampled.

We used differences in the modality of zone distributions to identify populations of isolates with distinct distinguished phenotypic resistant patterns. Where we classified isolates as resistant or susceptible, we used empirically derived custom susceptibility breakpoints, because upon examination of the distributions of the zones of inhibition the externally defined breakpoints based on wild-type cutoff values (1) did not accurately reflect the local population of *E. coli* strains. These custom breakpoints have limited clinical utility, but they improved our ability to distinguish between populations of bacteria in our sample. To define susceptibility breakpoints, we fitted two-component mixture models to zone diameter distributions of production bird isolates to index isolates into susceptible and resistant populations. We selected among three expectation maximum algorithms from R’s mixtools package (2): a parametric, semi-parametric and non-parametric specification that accounted for repeated measurement data to account for multiple isolates cultured from each sample. Among those, the best-fit model was selected based on the log-likelihood statistic. Custom breakpoints were set where the density estimates for the fitted distributions intersect, as described by (3). These updated breakpoints were rounded to the nearest whole number and used to categorize all isolates in our data as susceptible and resistant.

Distributions suggested a population of all resistant or all susceptible bacteria if Hartigans' dip statistic (which measures multimodality in a sample) had a *P*-value >0.1, failing to reject the null hypothesis of unimodality (4), or if the estimated proportion of a component in a mixture was lower than 0.01 (5). Otherwise, the distributions were considered bimodal, suggesting a mixture of both susceptible and resistant bacterial populations.

***Qualitative & Chemical Characterization of Poultry Feed***

Ethnographic interviews revealed that poultry feed comes from three local manufacturers (Pronaca, Agripac, and Nutril). Corn is the principal ingredient in all varieties, with additional protein from seafood added to accelerate growth. The ingredients of the feed listed unspecified "antibiotics." Guidelines from suppliers and veterinarians instruct villagers to add additional antibiotic and vitamin supplements to the animals' water on certain days. For example, Nutril's guidelines suggest application of unspecified "vitamins" on days 1-3, 8, 16-17, 22, 25, and "antibiotics" on days 9-10 and 23-24, as well as when a respiratory infection is noted. Several of the packets branded as "vitamins" for sale by veterinary supply stores have antibiotics listed in the ingredients (in particular, oxytetracycline, streptomycin, and enrofloxacin), suggesting that administration of vitamins may also introduce these antibiotics to the animals. There was no evidence that villagers were instructed in the practice of ceasing supplementation with antibiotics one week prior to slaughter. Villagers cited a wide variety of practices with respect to application of these supplements, with some applying more than the recommendations called for and others not supplementing their flock at all, usually due to financial constraints.

Mass spectrometry analysis detected antibiotics in all types of Nutril-brand feed sampled. Feed intended for broilers and laying hens contained virginiamycin, chloramphenicol and lincomycin. In addition, tetracycline was found in ground corn not formulated specifically as poultry feed (Supplemental Table S1). Our surveys determined that the majority of birds (75% of 81 flocks surveyed) were given Nutril-brand feeds. Two of the four antibiotics added to the feed (tetracycline and chloramphenicol) are active against *E. coli* and were included in our testing panel.

***Additional Ethnographic Details on Poultry Production Practices***

Villagers state convenience as a key reason for engaging in farming: the production cycle is highly integrated, with a single firm providing hatchery-raised animals and branded feed. Feed and additional supplies (antibiotic supplements and vaccines) are acquired from local veterinary pharmacies or development agency offices. Little training is required to engage in production.

Most farmers raise broiler chickens for meat production, with fewer households raising laying hens. Chicks are purchased from local veterinary stores, where they are given prophylactic antibiotics. The dominant broiler breed reached a maximum weight of 3.5-4 kg within eight weeks, but birds are usually slaughtered around week six as decreasing marginal weight gain after that point makes longer cycles less profitable. While there are different breeds of production birds, the variations are minor and we are unaware of any systematic differences in how sub-types of production birds are distributed and raised.

In contrast to production birds, household birds were raised for local consumption. Like production birds, villagers purchased household birds as chicks from the same veterinary stores, but the breeds are markedly different in appearance from broilers or laying hens. Veterinary stores vary in terms of administration practices, but in general chicks of the household varieties do not require antibiotics for prophylaxis: villagers state they are better suited to local conditions, and interviews with local farmers and veterinary store owners confirmed that household birds are rarely if ever given antibiotics. They are fed corn, yucca, coconut, and scraps of food instead of commercial feed.

**References**

1. **Wiggins B**. 1996. Discriminant analysis of antibiotic resistance patterns in fecal streptococci , a method to differentiate human and animal sources of fecal pollution in natural waters. Appl Environ Microbiol **62:**3997-4002.

2. **Benaglia T, Chauveau D, Hunter DR, Young DS**. 2009. mixtools: an R package for analyzing finite mixture methods. J Stat Softw **32**:1–29.

3. **Budczies J, Klauschen F, Sinn BV, Győrffy B, Schmitt WD, Darb-Esfahani S, Denkert C**. 2012. Cutoff Finder: a comprehensive and straightforward web application enabling rapid biomarker cutoff optimization. PLoS One **7:**e51862.

4. **Hartigan JA, Hartigan PM**. 1985. The dip test of unimodality. Ann Stat **13:**70-84.

5. **Díaz-Muñoz SL, Tenaillon O, Goldhill D, Brao K, Turner PE, Chao L**. 2013. Electrophoretic mobility confirms reassortment bias among geographic isolates of segmented RNA phages. BMC Evol Biol **13**:206.

6. **Clinical and Laboratory Standards Institute.** 2009. Performance standards for antimicrobial disk and dilution susceptibility tests for bacteria isolated from animals. Approved standard M31-A4 (vol. 28). Clinical and Laboratory Standards Institute, Wayne, PA.

7. **Clinical and Laboratory Standards Institute.** 2012. Performance standards for antimicrobial susceptibility testing; 22nd informational supplement. CLSI M100-S22. Clinical and Laboratory Standards Institute, Wayne, PA.
