# Supplementary material for: Antibiotic Resistance in Animal and Environmental Samples Associated with Small-Scale Poultry Farming in Northwestern Ecuador
Source: mSphere. 2016 Feb 10;1(1):e00021-15. doi: 10.1128/mSphere.00021-15 (PMC4863614; doi:10.1128/mSphere.00021-15)
Supplement: Figure S6 [file sph001162003sf10.docx]

**Supplemental Figure S6A**

**Supplemental Figure S6B**
