# Supplementary material for: Antibiotic Resistance in Animal and Environmental Samples Associated with Small-Scale Poultry Farming in Northwestern Ecuador
Source: mSphere. 2016 Feb 10;1(1):e00021-15. doi: 10.1128/mSphere.00021-15 (PMC4863614; doi:10.1128/mSphere.00021-15)
Supplement: Table S1 [file sph001162003st2.docx]

**Supplemental Table S1**

| **Type of animal** | **Type of feed** | **Result** |
| --- | --- | --- |
| Laying hen | Pre-initial laying hen feed | chloramphenicol |
| Laying hen | Laying hen feed | tetracycline, virginiamycin |
| Broiler chicken | Initial feed | virginiamycin |
| Broiler chicken | Initial feed | chloramphenicol, virginiamycin |
| Broiler chicken | Fattening/finishing feed | virginiamycin |
| Broiler chicken | Fattening/finishing feed | chloramphenicol, lincomycin |
| Any | Ground corn | tetracycline |
