# Supplementary material for: Antibiotic Resistance in Animal and Environmental Samples Associated with Small-Scale Poultry Farming in Northwestern Ecuador
Source: mSphere. 2016 Feb 10;1(1):e00021-15. doi: 10.1128/mSphere.00021-15 (PMC4863614; doi:10.1128/mSphere.00021-15)
Supplement: Table S2 [file sph001162003st3.docx]

**Supplemental Table S2**

|  | **Environment** | | **Poultry** | | **Total Samples** | **Total Isolates** |
| --- | --- | --- | --- | --- | --- | --- |
| **Site** | **Samples** | **Isolates** | **Samples** | **Isolates** | |  |
| **A (NA)** | 13 | 42 | 30 | 90 | 43 | 132 |
| Jul 2012 |  |  | 5 | 15 | 5 | 15 |
| Aug 2012 | 13 | 42 | 25 | 75 | 38 | 117 |
| **B (high-intensity)** | 114 | 278 | 36 | 107 | 150 | 385 |
| Nov 2010 |  |  | 17 | 50 | 17 | 50 |
| Jun 2011 | 100 | 227 |  |  | 100 | 227 |
| Mar 2012 | 14 | 51 | 9 | 27 | 23 | 78 |
| Mar 2013 |  |  | 10 | 30 | 10 | 30 |
| **C (low-intensity)** | 17 | 64 | 19 | 56 | 36 | 120 |
| Jan 2012 | 7 | 45 | 9 | 27 | 16 | 72 |
| Apr 2013 |  |  | 10 | 29 | 10 | 29 |
| **D (low-intensity)** | 14 | 50 | 30 | 87 | 44 | 137 |
| Nov 2010 |  |  | 10 | 30 | 10 | 30 |
| Apr 2012 | 14 | 50 | 10 | 28 | 24 | 78 |
| Jul 2013 |  |  | 10 | 29 | 10 | 29 |
| **E (low-intensity)** | 9 | 30 | 20 | 58 | 29 | 88 |
| Jan 2012 | 9 | 30 | 10 | 29 | 19 | 59 |
| Apr 2013 |  |  | 10 | 29 | 10 | 29 |
| **F (mid-intensity)** | 21 | 68 | 83 | 232 | 104 | 300 |
| Aug 2010 |  |  | 24 | 59 | 24 | 59 |
| Nov 2011 | 21 | 68 | 30 | 90 | 51 | 158 |
| Feb 2013 |  |  | 29 | 83 | 29 | 83 |
| **G (mid-intensity)** | 13 | 49 | 18 | 53 | 31 | 102 |
| May 2012 | 13 | 49 | 10 | 29 | 23 | 78 |
| Jun 2013 |  |  | 8 | 24 | 8 | 24 |
| **H (high-intensity)** | 89 | 216 | 130 | 365 | 219 | 581 |
| Aug 2010 |  |  | 33 | 84 | 33 | 84 |
| Jul 2011 | 73 | 164 |  |  | 73 | 164 |
| Nov 2011 | 16 | 52 | 45 | 122 | 61 | 174 |
| Feb 2013 |  |  | 52 | 159 | 52 | 159 |
| **I (mid-intensity)** | 15 | 48 | 19 | 55 | 34 | 103 |
| Jan 2012 | 15 | 48 | 9 | 27 | 24 | 75 |
| Apr 2013 |  |  | 10 | 28 | 10 | 28 |
| **J (high-intensity)** | 45 | 115 | 42 | 117 | 87 | 232 |
| Nov 2010 |  |  | 23 | 64 | 23 | 64 |
| Jun 2011 | 36 | 80 |  |  | 36 | 80 |
| Mar 2012 | 9 | 35 | 10 | 30 | 19 | 65 |
| Mar 2013 |  |  | 9 | 23 | 9 | 23 |
| **K (low-intentisy)** | 10 | 35 | 25 | 75 | 35 | 110 |
| Aug 2010 |  |  | 5 | 15 | 5 | 15 |
| Nov 2011 | 10 | 35 | 10 | 30 | 20 | 65 |
| Feb 2012 |  |  | 10 | 30 | 10 | 30 |
| **L (mid-intensity)** | 18 | 53 | 61 | 179 | 79 | 232 |
| Nov 2010 |  |  | 22 | 64 | 22 | 64 |
| Apr 2012 | 18 | 53 | 19 | 55 | 37 | 98 |
| Jul 2013 |  |  | 20 | 60 | 20 | 60 |
| **M (high-intensity)** | 56 | 155 | 39 | 114 | 95 | 269 |
| Jul 2011 | 38 | 96 |  |  | 38 | 96 |
| May 2012 | 18 | 59 | 19 | 57 | 37 | 116 |
| Jun 2013 |  |  | 20 | 57 | 20 | 57 |
| **N (NA)** | 16 | 44 | 27 | 70 | 43 | 114 |
| Nov 2010 |  |  | 7 | 18 | 7 | 18 |
| Apr 2012 | 16 | 44 | 10 | 26 | 26 | 70 |
| Jul 2013 |  |  | 10 | 26 | 10 | 26 |
| **O (high-intensity)** | 67 | 155 | 28 | 96 | 95 | 251 |
| Jul 2011 | 54 | 117 |  |  | 54 | 117 |
| May 2012 | 13 | 38 | 9 | 29 | 22 | 67 |
| Jun 2013 |  |  | 19 | 67 | 19 | 67 |
| **P (low-intensity)** | 13 | 46 | 9 | 23 | 22 | 69 |
| Jan 2012 | 13 | 46 | 9 | 23 | 22 | 69 |
| **Q (mid-intensity)** | 9 | 31 | 35 | 98 | 44 | 129 |
| Aug 2010 |  |  | 5 | 12 | 5 | 12 |
| Nov 2011 | 9 | 31 | 10 | 30 | 19 | 61 |
| Feb 2013 |  |  | 20 | 56 | 20 | 56 |
| **Grand Total** | **529** | **1460** | **651** | **1875** | **1190** | **3354** |
