# Supplementary material for: Antibiotic Resistance in Animal and Environmental Samples Associated with Small-Scale Poultry Farming in Northwestern Ecuador
Source: mSphere. 2016 Feb 10;1(1):e00021-15. doi: 10.1128/mSphere.00021-15 (PMC4863614; doi:10.1128/mSphere.00021-15)
Supplement: Table S3 [file sph001162003st4.docx]

**Supplemental Table S3**

| **Antibiotic** | **CLSI breakpoint range, Resistant–Susceptible, mm** | **Custom breakpoint, mm** | **% Non-susceptible, CLSI susceptible BPs (*N*=3860 isolates)** | **% Non-susceptible, custom BPs (*N*=3860 isolates)** |
| --- | --- | --- | --- | --- |
| **Amoxicillin/clavulanate** | 13–18 | 14.62 | 12.77% | 7.89% |
| **Ampicillin** | 13–17 | 12.38 | 28.22% | 23.06% |
| **Cefotaxime** | 22–26 | 25.59 | 10.22% | 10.19% |
| **Cephalothin** | 14–18 | 9.4 | 60.84% | 8.34% |
| **Chloramphenicol** | 12–18 | 15.8 | 12.41% | 11.90% |
| **Ciprofloxacin** | 15–21 | 18.2 | 12.44% | 11.03% |
| **Enrofloxacin** | 16–23 | 15.35 | 24.11% | 10.94% |
| **Gentamicin** | 12–15 | 13.17 | 6.06% | 5.40% |
| **Streptomycin** | 11–15 | 8.51 | 39.43% | 14.06% |
| **Sulfisoxazole** | 12–17 | 12.83 | 34.36% | 32.83% |
| **Tetracycline** | 11–15 | 14.33 | 43.33% | 43.30% |
| **Trimethoprim** | 10–16 | 13.86 | 29.78% | 29.57% |
